# Supplementary material for: Polyketide synthase gene clusters in the ericoid mycorrhizal fungus Oidiodendron maius: genome-wide prediction, expression profiling, and functional characterization of knockout mutants
Source: Front Plant Sci. 2026 Sep 16;17:1924430. doi: 10.3389/fpls.2026.1924430 (PMC13623903; doi:10.3389/fpls.2026.1924430)
Supplement: Supplementary file 1 [file SupplementaryFile1.zip › Supplementary Materials/Supplementary Folder 1.zip!/Oidma1_AssemblyScaffolds_Repeatmasked/knownclusterblast/cluster2/jgi.p_Oidma1_36643_mibig_hits.html]

| MIBiG Protein | Description | MIBiG Cluster | MiBiG Product | % ID | % Coverage | BLAST Score | E-value |
| --- | --- | --- | --- | --- | --- | --- | --- |
| AEF32090.1 | monooxygenase | BGC0001333 | BE-54017\_biosynthetic\_gene\_cluster | 26 | 82.5613079019 | 79 | 9.1e-15 |
| AAM33676.1 | putative\_FAD-dependent\_oxidoreductase\_GrhO9 | BGC0000230 | Griseorhodin\_biosynthetic\_gene\_cluster | 28 | 85.8310626703 | 79 | 1.2e-14 |
| AEO12714.1 | flavin\_monooxygenase | BGC0001334 | Cladoniamide\_biosynthetic\_gene\_cluster | 25 | 84.4686648501 | 69 | 9.4e-12 |
| AHX24715.1 | FAD-binding\_monooxygenase | BGC0000200 | Arixanthomycin\_biosynthetic\_gene\_cluster | 25 | 84.4686648501 | 69 | 1.2e-11 |
| AKQ20687.1 | monooxygenase | BGC0001337 | Lazarimide\_biosynthetic\_gene\_cluster | 26 | 83.10626703 | 67 | 2.7e-11 |
| BAJ52699.1 | putative\_FAD-binding\_monooxygenase | BGC0000222 | FD-594\_biosynthetic\_gene\_cluster | 24 | 85.0136239782 | 62 | 8.8e-10 |
| ABV97173.1 | monooxygenase\_FAD-binding | BGC0000137 | Rifamycin\_biosynthetic\_gene\_cluster | 26 | 81.4713896458 | 59 | 1.3e-08 |
